# ABO phenotype and clinical correlates of COVID-19 severity in hospitalized patients

**DOI:** 10.2144/fsoa-2021-0045

**Published:** 2021-06-12

**Authors:** David J Hermel, Samantha R Spierling Bagsic, Carrie L Costantini, James R Mason, Zhubin J Gahvari, Alan Saven

**Affiliations:** ^1^Division of Hematology & Oncology, Scripps Clinic, La Jolla, CA 92037, USA

**Keywords:** ABO blood type, anemia, COVID-19, Hispanic, male, San Diego

## Abstract

**Aim:** This study investigates the association between ABO blood phenotype and COVID-19 severity, measured by intensive care unit admission, need for intubation, hospitalization length and death. It further explores clinical predictors of COVID-19 severity within a primarily Hispanic demographic in San Diego County. **Materials & methods:** We retrospectively reviewed 942 total patients, 473 with available blood type, hospitalized at five Scripps Health hospitals with COVID-19. **Results:** No significant association was found between ABO phenotype and COVID-19 severity on multivariate analysis, while a diagnosis of anemia and male sex was associated with all severity outcomes on exploratory analysis. **Conclusion:** Our results provide relevant clinical correlates of COVID-19 severity and help better elucidate the association between ABO phenotype and COVID-19.

## Lay abstract

In patients diagnosed with COVID-19, identifying those at increased risk for morbidity and mortality is imperative given the wide spectrum of clinical disease manifestations. Emerging evidence supports an association between ABO blood phenotype and COVID-19 disease susceptibility, with additional studies suggesting that certain blood types may augment and/or suppress disease progression. We retrospectively reviewed 942 hospitalized patients in San Diego County with COVID-19 and found no significant association between ABO blood group type and severity outcomes after accounting for confounding variables. Furthermore, a diagnosis of anemia and male sex was universally associated with all COVID-19 severity outcomes on exploratory analyses.

Review of the clinical characteristics, laboratory findings and outcomes of patients with COVID-19 has elucidated distinct patterns of disease and unique biological risk factors for severe illness and death. Identifying high-risk clinical features and diagnostic predictors of morbidity and mortality remains imperative. To date, large epidemiological studies of patients with COVID-19 have shown that the presence of specific comorbid medical conditions – including diabetes mellitus, hypertension and respiratory and cardiovascular disease [1] – as well as older age [2], obesity [3], male sex [4] and immunosuppression [5] are all important risk factors for disease severity and death.

Building upon these established clinical risk factors, detailed analyses of large-scale patient data have suggested an important association between ABO blood group phenotype and vulnerability to infection by SARS-CoV-2. Initial observational studies from China [6–8] that were subsequently duplicated globally [9–13] have noted a decreased incidence of COVID-19 in patients with type O blood relative to the regional, study-specific population under investigation. However, whether blood type O or other ABO blood groups alter the clinical course of COVID-19 after infection is unclear, with variable results in different study populations [10–12,14–19].

To test whether an association exists between ABO phenotype and COVID-19 severity in our San Diego County study population, we conducted a large, retrospective review of patients hospitalized for COVID-19, factoring in relevant demographic and clinical confounders. Moreover, we sought to identify any potential clinical or demographic determinants of severe COVID-19 infection, defined as admission to the intensive care unit (ICU), intubation, length of hospital stay (LOS) or death.

## Materials & methods

All patients hospitalized within the regional Scripps Health hospital system in San Diego County from 1 March 2020 to 1 September 2020 with a diagnosis of COVID-19 were included in the initial dataset. COVID-19 was confirmed by the testing platforms in use at Scripps Medical Laboratories, including the Hologic and Abbott ID Now SARS-CoV-2 PCR assays. The threshold of positivity was standardized across Scripps laboratories at different hospitals using the same universal US FDA approved testing platforms with concordant testing treatment algorithm. Only patients who were hospitalized with a positive COVID-19 test during or within 1 day of hospital admission were included in the final analysis. Individuals meeting these inclusion criteria with multiple hospitalizations were included only for the initial hospitalization following a positive COVID test. Patients with do-not-resuscitate orders were not excluded from this analysis. Relevant laboratory and clinical data were extracted from the Scripps Health electronic medical record. Only patients with available ABO blood type data were included in primary outcome analyses with blood type as a variable. ABO blood type may have been tested for in a prior hospitalization or outpatient visit and/or the current admission encounter at clinician discretion. In addition to ABO blood type stratification, all patients were included in exploratory multivariable analyses that did not just include blood type.

Clinical information extracted included demographic details, ABO blood type, Rhesus (Rh) factor positivity and baseline clinical comorbidities. Demographic details included age, BMI, sex and ethnicity (grouped as either Hispanic or non-Hispanic). Patient comorbidities of interest were determined based on the International Classification of Diseases Codes, Ninth and Tenth Revision (ICD-9/10 codes) listed within each patients’ medical record during the hospitalization of interest.

All laboratory results and medications administered within a patient’s hospitalization were extracted from the electronic medical record. Medications of interest included steroids, anticoagulants, vasopressors, remdesivir, azithromycin, hydroxychloroquine, statins, proton pump inhibitors and H2-receptor antagonists.

Clinical severity outcomes of interest included admission to the ICU, intubation during hospitalization, LOS and death. Death was defined as any mortality within the study period.

### Statistical methods

Individuals meeting inclusion criteria (n = 942), with blood type on file (n = 473), were grouped by blood type (A, B, AB, O) for all outcome analyses. Demographic clinical characteristics, comorbid conditions, labs of interest and medication were compared between blood type groups. Categorical variables were presented as frequencies and percentages and were compared between groups using Pearson’s chi-Square test, and if there was a significant overall effect, to discern which groups significantly differed, posthoc pairwise chi-Square tests with Holm’s adjustment were conducted if there was an overall significant association of blood type with the variable of interest. Continuous variables were presented as means and standard deviations and were compared first by one-way analysis of variance (ANOVA) to identify any overall effects of blood type and if significant, to discern which groups significantly differed, posthoc pairwise Tukey’s tests were conducted to discern differences. Outcome variables are presented as frequencies and proportions as well as calculated odds ratios and 95% CI, relative to blood type A. Logistic regression was employed to test whether blood type was associated with any of the study outcomes (ICU stay, intubation and death) with blood type A being treated as the reference condition. Follow-up multivariable logistic regression analyses were determined for each outcome that included demographic and comorbidity variables that were independently associated with blood type to test for conditional effects. Blood type was also tested as an independent predictor in a Cox proportional hazards model on time to discharge with censoring on in-hospital death. Hazards ratios and 95% CI were calculated relative to blood type A. Median LOS regardless of outcome (alive vs deceased) was also compared by a Kruskal–Wallis test. Exploratory multivariable models were conducted for each study outcome (ICU stay, intubation and death) starting with any demographic, clinical characteristic or comorbidity variables that were significant in independent univariable analyses and following a backward elimination method to reduce to a final model. All analyses were conducted in R v.3.5.3.

## Results

In total, 473 patients had an available blood type in the medical record. Overall, 143 (30.23%) were blood type A, 267 (56.45%) were blood type O, 45 (9.51%) were blood type B and 18 (3.81%) were blood type AB. About 439 patients (92.81%) were Rh positive.

### Demographic & clinical characteristics

Please see Table 1 for significant (p < 0.05) demographic and clinical characteristics stratified across different ABO subgroups. The average age of the entire cohort was 61.23 ± 16.70 years, mean BMI was 30.88 ± 8.80, 72.52% were Hispanic and 55.18% were male. Patients with blood type B were, on average, younger than those with blood type A (55.73 ± 18.05 vs 64.2 ± 16.51 years; p = 0.015). The distribution of ethnicity differed overall among blood types (p = 0.006), but pairwise comparisons did not reach statistical significance.

**Table 1.** Demographic and clinical characteristics that differ across ABO blood type groups.

Blood type (sample size)	A (143)	AB (18)	B (45)	O (267)	Main effect^†^	Pairwise *post hoc* comparisons (p-values)					
Demographics	n (%)	n (%)	n (%)	n (%)	p-value	A-AB	A-B	A-O	AB-B	AB-O	B-O
Hispanic ethnicity	102 (71.3%)	9 (50%)	26 (57.8%)	206 (77.2%)	0.006	0.463	0.463	0.476	0.779	0.105	0.061
Age^§^, mean (SD)	64.2 (16.5)	64.83 (20.3)	55.73 (18.1)	60.32 (16.0)	0.011	0.999	0.015	0.110	0.200	0.678	0.315

**Comorbidities**	**n (%)**	**n (%)**	**n (%)**	**n (%)**	**p-value**	**A-AB**	**A-B**	**A-O**	**AB-B**	**AB-O**	**B-O**
CKD or ESRD	37 (25.9%)	9 (50%)	7 (15.6%)	69 (25.8%)	0.047	0.255	0.581	>0.999	0.071	0.255	0.581

**Initial lab values**^‡^	**Mean (SD)**	**Mean (SD)**	**Mean (SD)**	**Mean (SD)**	**p-value**	**A-AB**	**A-B**	**A-O**	**AB-B**	**AB-O**	**B-O**
Peak creatinine^¶‡^ (Cr, 0.5–1.0 mg/dl)	2.06 (2.29)	3.8 (3.53)	1.4 (1.73)	2.28 (3.09)	0.018	0.062	0.513	0.872	0.012	0.115	0.205
Peak neutrophils^#‡^ (neutrophils %, 38–74%)	83.4 (9.8)	88.52 (4.71)	81.69 (8.9)	84.96 (9.13)	0.020	0.134	0.696	0.360	0.046	0.411	0.123
Mean lymphocytes^#‡^ (lymphocytes %, 16–48%)	14.35 (7.7)	11.06 (4.38)	17.61 (8.46)	14.94 (8.02)	0.019	0.364	0.074	0.886	0.019	0.200	0.154
Monocytes^#^ (monocytes %, 4.9–12.5%)	6.97 (3.99)	5.29 (2.97)	6.03 (2.77)	6.02 (3.42)	0.038	0.248	0.406	0.048	0.881	0.841	>0.999
Peak D-dimer (<500 ng/ml)^††‡^	2394.68 (2755.2)	3342.95 (3034.84)	1491.12 (1994.32)	2687.8 (2799.17)	0.040	0.559	0.260	0.775	0.099	0.789	0.049

† Main effect p-values indicate overall comparison across all blood type groups. Only significant main effects have respective pairwise comparisons with p-values shown.

‡ Specified measures were not from initial labs and represent either peak or mean across entire hospitalization.

§ One patient missing data, n = 472 for age.

¶ Metabolic panel n = 464–465.

# Blood count n = 471–473.

†† Inflammatory markers n = 395–408.

CKD: Chronic kidney disease; ESRD: End-stage renal disease; SD: Standard deviation.

In addition, within this cohort, the specific prevalence of comorbidities was noteworthy with 62.16% hypertension, 58.35% diabetes mellitus, 38.69% hyperlipidemia (HLD), 25.79% end-stage renal disease (ESRD) or chronic kidney disease (CKD), 20.08% atrial fibrillation, 18.82% with deep venous thrombosis (DVT) and/or pulmonary embolism (PE), 14.80% chronic obstructive pulmonary disease and 13.74% cancer. Of these comorbid conditions, only CKD/ESRD incidence differed among blood groups (p = 0.047), but pairwise comparisons did not reach statistical significance.

### Laboratory results

Table 1 additionally includes significant laboratory blood tests among ABO stratified patients. Mean laboratory values on hospital admission were remarkable for an elevated aspartate aminotransferase (AST) and alanine transaminase (ALT) (72.66 and 53.08 units/l, respectively), hyponatremia (sodium 137.17 mmol/l), an elevated serum creatinine (1.73 mg/dl) and increased inflammatory markers including CRP (130.28 mg/l), D-dimer (1570.46 ng/ml) and procalcitonin (2.28 ng/ml) in those undergoing testing for these markers. There were no major differences in baseline laboratory values among ABO subgroups on admission, with a slightly, but significantly, higher monocyte percentage in patients with type A compared with type O blood (6.97 ± 3.99 vs 6.02 ± 3.42%; p = 0.048).

Analysis of the peak, nadir and mean of all laboratory values of interest were compared among ABO stratified patients over the course of hospitalization. The statistically significant differences were a decreased peak serum creatinine in patients with type B compared with type AB blood (1.4 ± 1.73 vs 3.8 ± 3.53 mg/dl; p = 0.012). Additionally, peak quantitative D-dimer was significantly decreased in type B compared with type O blood (1491.12 ± 1994.32 vs 2687.80 ± 2799.17 ng/ml; p = 0.049). White blood cell (WBC) subset analysis revealed a decreased peak neutrophil percentage in patients with type B compared with type AB blood (81.69 ± 8.9 vs 88.52 ± 4.71%; p = 0.046) and an increased mean hospital lymphocyte percentage in patients with type B compared with type AB blood (17.61 ± 8.46 vs 11.06 ± 4.38 K/mcl; p = 0.019).

### Medications

With regards to in-hospital administered medications, 68.92% received steroids, 49.89% remdesivir, 45.88% azithromycin, 37.63% vasopressors and 89.01% anticoagulants. There were no significant differences in the medications of interest received among the different blood groups.

### Severity outcomes

**Figure 1.**
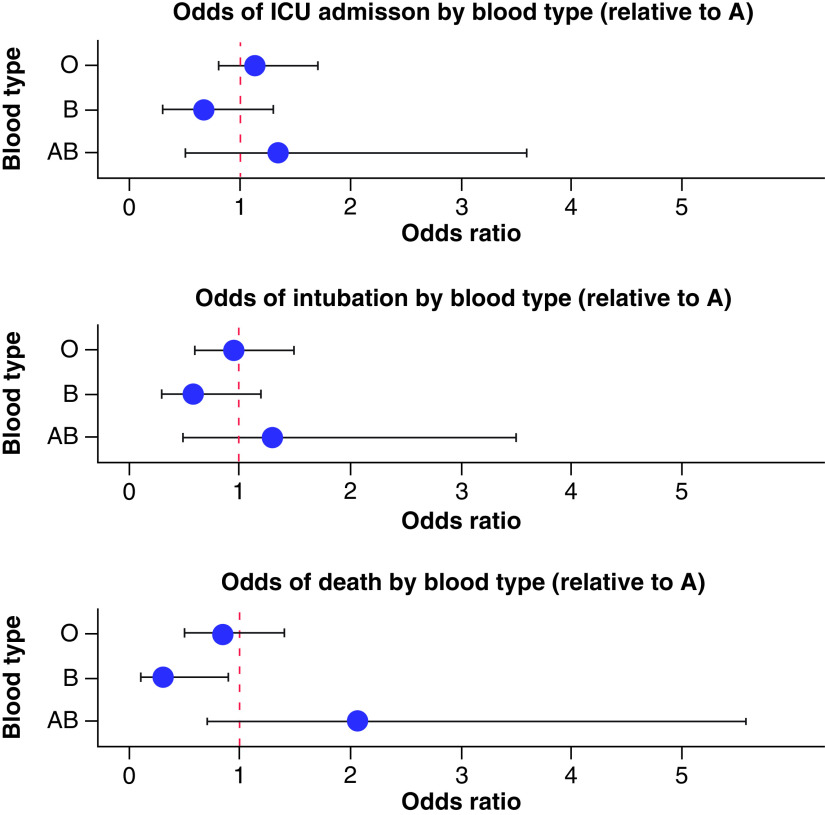
Odds ratio plots for primary outcomes by blood type. Odds of ICU admission, intubation and death for blood types O, B and AB relative to blood type A. The odds of death was lower among blood type B relative to blood type A on univariate analysis. ICU: Intensive care unit.

In total, among hospitalized patients stratified according to ABO phenotype, 207 patients (43.76%) were admitted to the ICU, 149 (31.50%) were intubated and 100 (21.14%) died. The median LOS was 10 days (IQR: 5–20). On primary outcome univariate analysis, there was no statistical significance between blood groups and ICU stay, LOS or intubation. However, there was a decreased odds of death in patients with type B blood (odds ratio [OR]: 0.31; 95% CI: 0.09–0.85, see Figure 1), yet this did not reach statistical significance after adjusting for variables that differed between ABO blood groups, including age, ethnicity and CKD/ESRD. Table 2 provides outcomes data.

**Table 2.** Study outcomes by blood group.

Outcomes	A (n = 143)		AB (n = 18)		B (n = 45)		O (n = 267)		Main effect	p-value (A is reference)			Adj^‡^ p-value
	n (%)	OR^†^ (95% CI)	n (%)	OR^†^ (95% CI)	n (%)	OR^†^ (95% CI)	n (%)	OR^†^ (95% CI)	p-value^†^	AB	B	O	
ICU	61 (42.7%)	Ref	9 (50%)	1.34 (0.5–3.6)	15 (33.3%)	0.67 (0.3–1.3)	122 (45.7%)	1.13 (0.8–1.7)	0.424				0.643
Intubation	47 (32.9%)	Ref	7 (38.9%)	1.3 (0.5–3.5)	10 (22.2%)	0.58 (0.3–1.2)	85 (31.8%)	0.95 (0.6–1.5)	0.477				0.590
Death	34 (23.8%)	Ref	7 (38.9%)	2.04 (0.7–5.6)	4 (8.9%)	0.31 (0.1–0.9)	55 (20.6%)	0.83 (0.5–1.4)	0.037	0.172	0.038	0.457	0.197

	Median^§^ (IQR)	HR^¶^ (95% CI)	Median^§^ (IQR)	HR^¶^ (95% CI)	Median^§^ (IQR)	HR^¶^ (95% CI)	Median^§^ (IQR)	HR^¶^ (95% CI)	LOS^§^p-value	Surv^¶^p-value	Adj^#^p-value		
LOS^#^	9 (5.0–17.0)	Ref	9 (5.3–15.3)	0.66 (0.4–1.2)	8 (4.0–17.0)	1.42 (1.0–2.0)	10 (6.0–21.0)	0.95 (0.8–1.2)	0.139	0.0695	0.1446		

† OR and 95% CI calculated from independent, univariable logistic regression to identify effects of blood type on ICU admissions, intubation and death.

‡ Adjusted p-values are from tests of conditional associations of blood type on each outcome in a multivariable logistic regression model including Hispanic ethnicity, age and CKD.

§ Median LOS was compared using a Kruskal–Wallis test.

¶ HR and 95% CI’s result from a univariable Cox proportional hazards model with blood type as an independent predictor of survival to discharge, censoring on in-hospital death, with p-value indicated.

# The adjusted p-value is from a multivariable Cox proportional hazards model to test conditional associations of blood type accounting for Hispanic ethnicity, age and CKD.

CKD: Chronic kidney disease; HR: Hazard ratio; ICU: Intensive care unit; IQR: Interquartile range; LOS: Length of hospital stay; OR: Odds ratio.

### Exploratory correlates of outcomes

Further exploratory clinical correlates of COVID-19 severity outcomes in the total 942 patients are included in Table 3. A diagnosis of anemia was significant, conditionally associated with ICU admission (OR: 2.76), intubation (OR: 5.04) and death (OR: 1.74). Those with a diagnosis of DVT and/or PE had a significantly elevated risk of admission to the ICU (OR: 2.62) and intubation (OR: 2.48), but not death. Male sex was universally associated with increased odds for all COVID-19 severity outcomes.

**Table 3.** Exploratory multivariable analyses for COVID-19 severity outcomes among all patients.

ICU admission	OR (95% CI)	p-value
Hispanic	1.69 (1.06–2.71)	0.0274
Sex	2.13 (1.42–3.22)	0.0003
Diabetes	1.88 (1.25–2.86)	0.0028
DVT/thrombosis/pulmonary embolism	2.62 (1.54–4.52)	0.0004
Anemia	2.76 (1.83–4.19)	<0.00001

**Intubation**	**OR (95% CI)**	**p-value**
Hispanic	1.96 (1.15–3.42)	0.0145
Sex	2.09 (1.33–3.33)	0.0017
BMI	1.03 (1.01–1.06)	0.0162
DVT/thrombosis/pulmonary embolism	2.48 (1.46–4.24)	0.0008
COPD/emphysema	2.18 (1.19–3.98)	0.0112
Anemia	5.04 (3.16–8.19)	<0.00001

**Death**	**OR (95% CI)**	**p-value**
Age	1.04 (1.03–1.06)	<0.00001
Sex	2.75 (1.67–4.63)	0.0001
COPD	1.93 (1.07–3.47)	0.0276
Anemia	1.74 (1.07–2.85)	0.0271

COPD: Chronic obstructive pulmonary disease; DVT: Deep venous thrombosis; OR: Odds ratio.

## Discussion

The broad range of clinical symptoms associated with SARS-CoV-2 infection has led to a thorough investigation into the biology of this viral illness as well as concomitant host factors, including ABO phenotype, that may influence disease morbidity and mortality. Results from this large, single-institution, multihospital retrospective evaluation of hospitalized COVID-19 patients in San Diego County do not show an association between ABO blood group type and COVID-19 disease severity. While there were lower odds of mortality in patients with type B blood relative to type A, this was not statistically significant after accounting for this cohort’s combined younger age, lower incidence of kidney dysfunction and variation in Hispanic ethnicity. On exploratory analyses, anemia and male sex were universally associated with all severity outcomes and diagnoses of DVT and/or PE were associated with intubation and ICU admission, but not death.

There is a compelling biologic rationale to support an association between ABO blood type and COVID-19 disease severity. For one, ABO peptides, which are expressed on endothelial cells and platelets, can influence susceptibility to thrombosis via interactions with von Willebrand factor and factor VIII [20] and thus can potentially augment the risk of consumptive coagulopathy and thrombotic microangiopathy in patients with COVID-19. In addition, the A and B carbohydrate structures are also expressed on epithelial cells in the gastrointestinal and respiratory tracts and, during SARS-CoV-2 replication in the epithelium, can be fully incorporated into the viral spike glycoprotein [21,22]. This potentially enables the virus to be neutralized by anti-A and/or anti-B antibodies, which can prevent viral cell entry and associated pathogenicity. Furthermore, ABO blood group antigens have been shown to modulate the innate immune response to various pathogens through their glycan moieties [23] and variants in the *ABO* gene group locus have been shown in a large European study to correlate with the development of respiratory failure in patients with COVID-19 [14]. Moreover, *ABO* gene polymorphisms are independently associated with cardiovascular disease [24], angiotensin converting enzyme activity [25], red blood cell indices [26] and venous thromboembolism [27], all of which have been reported to serve a pivotal role in the pathogenesis of severe COVID-19 infection [28].

In contradistinction to other clinical studies, the study population investigated encompasses a large Hispanic demographic in the Southwestern region of the USA. Consistent with Hispanic variation in blood type, the most prevalent blood type was type O and the vast majority of patients were Rh positive [29]. While overt difference in ABO gene allelic frequency are recognized among Hispanics, additional differences in this population include variations in minor antigens that are unaccounted for and may be important in influencing outcome measures [30]. Though ABO group distribution significantly differed among Hispanic and non-Hispanic ethnicity, there was still a significantly decreased risk of death (p = 0.0418) in patients with type B blood relative to type A after accounting for Hispanic ethnicity as a predictor of death. Among the Hispanic population, there was a significant difference in the distribution of ABO blood groups (p = 0.048), but there were only nine patients with AB blood types limiting cross comparison. Within this hospitalized population, Hispanic ethnicity was conditionally associated with both admission to the ICU and need for intubation, reflecting the disproportionate morbidity in this highly represented patient demographic.

Though nonsignificant after multivariate analysis, these results indicate a possible link between blood type B and decreased mortality risk, which has been previously described [10,11]. It is of interest to note that over the course of hospitalization patients with blood type B had the lowest peak serum D-dimer levels, the lowest peak creatinine levels, the lowest peak neutrophil percentages and the highest mean lymphocyte percentages. Despite not identifying significant differences in absolute WBC counts between groups, decreased absolute lymphocytes [31], elevated D-dimer [32], increased absolute neutrophils [33] and increased creatinine [33] have been shown to reflect clinical disease severity and/or fatality in COVID-19. The marginal decreased odds of death in blood type B, as well as the stark differences in laboratory values in this subgroup compared with blood type AB, are consistent with a possible mechanistic role for anti-A isoagglutinins in the pathogenesis of severe COVID-19 and previously described ability to disrupt the interaction between SARS-CoV-2 and the host angiotensin converting enzyme-2 receptor [22,34]. However, no mortality improvement in type O blood with similar anti-A antibodies was identified, possibly because the immunoglobulin predominant isotype is often IgG rather than IgM in type O blood [22,34]. However, the most important predictor of death in our cohort was age and the age in blood group B patients was markedly lower than other blood groups. Thus, in analyses of significant blood type variation controlling for age, the differences observed among blood types were not significant.

Complementing this ABO stratified data analysis, exploratory analysis of all patients hospitalized with COVID-19 at these five hospitals found that male sex was significantly associated with an increased risk of ICU admission, intubation and death. Associations between gender and outcome have been previously reported and were thought to be due to hormone-regulated expression of viral entry receptors and differences in immune responses between sexes [35]. Likewise, a diagnosis of anemia was found to be a conditional correlate for death, intubation and ICU admission, which may reflect the underlying pathophysiology as well as the manifestations of an anemic state. In addition, those with a diagnosis of either a DVT and/or PE had a higher incidence of ICU admission and intubation, but this did not correlate with death, possibly reflected in the high-rate of anticoagulant use.

Some possible limitations of this study are that it is an observational, retrospective study with inherent confounding and lead-time bias. Sample size was relatively small and prevalence of each blood type was not uniform, with a particularly low sample size in the AB blood type. Patients without a documented blood type were excluded from the primary analysis. We only included the first admission immediately following a COVID-19 diagnosis and excluded multiple re-admissions from analyses, so it is possible individuals not reported to have an outcome of interest during this hospitalization could have had such an outcome in future re-hospitalization(s). Additionally, more severely affected patients may have been more likely to have ABO blood grouping performed as a prelude to a potential blood product transfusion. Moreover, patients with known ABO groups had significantly higher rates of anemia and likely required more blood transfusions than nontyped patients.

This comprehensive, demographically unique study adds to the growing body of literature on this topic and furthers important insight into the association between ABO blood type and COVID-19. Larger international collaborative studies are warranted to further elucidate the relationship between COVID-19 and ABO blood type, particularly in diverse populations.

## Future perspective

Given the stark heterogeneity in COVID-19 outcomes among patients, it is critical to assess for predictors of disease severity to optimally risk-stratify infected patients. As more data are accumulated regarding patient outcomes, important associations between disease severity and clinical risk factors will be better refined. With large collaborative multi-institutional, international data sets, we can better explore the association between ABO blood type as well as other clinical factors with COVID-19 severity.

**Summary points**

- We conducted a retrospective, observational study of patients hospitalized within our regional five hospital network system in San Diego county to investigate an association between ABO/Rh blood type and COVID-19 disease severity.
- In our study, we found no significant association between ABO phenotype and COVID-19 disease morbidity, including admission to the intensive care unit, intubation or length of hospital stay. However, there was a marginal decreased odds of death in patients with type B blood, though this was not significant after adjusting for confounding variables that differed between blood types, including age, ethnicity and underlying kidney disease.
- In exploratory analyses assessing clinical correlates of COVID-19 severity in our patient cohort, we found that a diagnosis of anemia was significantly, conditionally associated with intensive care unit admission, intubation and death. In addition, those with a diagnosis of deep venous thrombosis and/or pulmonary embolism had an elevated risk of COVID-19 morbidity but not mortality, possibly related to widespread prophylactic anticoagulation use. Male sex was universally associated with all COVID-19 severity outcomes.
